# Lifestyle domains as determinants of wheeze prevalence in urban and rural schoolchildren in Ecuador: cross sectional analysis

**DOI:** 10.1186/1476-069X-14-15

**Published:** 2015-02-04

**Authors:** Alejandro Rodriguez, Maritza G Vaca, Martha E Chico, Laura C Rodrigues, Mauricio L Barreto, Philip J Cooper

**Affiliations:** Laboratorio de Investigación FEPIS, Quinindé, Esmeraldas Province Ecuador; Centro de Investigación en Enfermedades Infecciosas y Crónicas, Pontificia Universidad Católica del Ecuador, Quito, Ecuador; Faculty of Epidemiology and Population Health, London School of Hygiene and Tropical Medicine, London, UK; Instituto de Saude Coletiva, Universidade Federal da Bahia, Salvador, Brazil; Clinical Sciences, St George’s University of London, London, UK

**Keywords:** Lifestyle domains, Wheeze, Schoolchildren, Urban, Rural, Tropics, Latin America

## Abstract

**Background:**

The acquisition of a modern lifestyle may explain variations in asthma prevalence between urban and rural areas in developing countries. However, the effects of lifestyle on asthma have been investigated as individual factors with little consideration given to the effects of lifestyle as a set of attributes. The aim of the present study was to identify modern lifestyle domains and assess how these domains might explain wheeze prevalence in urban and rural areas.

**Methods:**

We analysed data from cross-sectional studies of urban and rural schoolchildren in Esmeraldas Province, Ecuador. Variables were grouped as indicators of socioeconomic factors, sedentarism, agricultural activities and household characteristics to represent the main lifestyle features of the study population. We used multiple correspondence analyses to identify common lifestyle domains and cluster analysis to allocate children to each domain. We evaluated associations between domains and recent wheeze by logistic regression.

**Results:**

We identified 2–3 lifestyle domains for each variable group. Although wheeze prevalence was similar in urban (9.4%) and rural (10.3%) schoolchildren, lifestyle domains presented clear associations with wheeze prevalence. Domains relating to home infrastructure (termed transitional, rudimentary, and basic urban) had the strongest overall effect on wheeze prevalence in both urban (rudimentary vs. basic urban, OR = 2.38, 95% CI 1.12-5.05, p = 0.024) and rural areas (transitional vs. basic urban, OR = 2.02, 95% CI 1.1-3.73, p = 0.024; rudimentary vs. basic urban, OR = 1.88, 95% CI 1.02-3.47, p = 0.043). A high level of sedentarism was associated with wheeze in the rural areas only (OR = 1.64, 95% CI 1.23-2.18, p = 0.001).

**Conclusions:**

We identified lifestyle domains associated with wheeze prevalence, particularly living in substandard housing and a high level of sedentarism. Such factors could be modified through programmes of improved housing and education. The use of lifestyle domains provides an alternative methodology for the evaluation of variations in wheeze prevalence in populations with different levels of development.

**Electronic supplementary material:**

The online version of this article (doi:10.1186/1476-069X-14-15) contains supplementary material, which is available to authorized users.

## Introduction

Secular trends of increasing prevalence of childhood asthma have been documented worldwide [1]. This observation, in parallel with differences in asthma prevalence between developed and developing countries and between urban and rural areas, has led to the suggestion that alterations in environmental and lifestyle factors are the most plausible explanation for such trends [2]. There is evidence also that asthma prevalence may have reached a plateau in developed countries in contrast with many developing regions where the prevalence of asthma continues to increase [3].

Asthma prevalence varies considerably between countries and may be lower in rural populations [3, 4]. The lower prevalence in rural populations could be explained by farming exposures associated with a traditional lifestyle that may confer protection against allergic diseases [5]. The prevalence of asthma appears to be increasing in urban and rural populations in developing countries [6, 7], and increasing urbanization and the acquisition of modern lifestyle, coupled perhaps with environmental triggers for non-atopic symptoms and or the persistence of exposures that attenuate atopy [8, 9], has been the most cited factors to explain these epidemiological trends [10, 11].

The most common lifestyle factors associated with the increase in asthma related to the process of urbanization are dietary changes, economic development, sedentary habits, changes in housing and a decline in farming activities, among others [12]. Commonly, the association between lifestyle factors and asthma has been examined in terms of the independent effects of individual risk factors, and such analyses can explain asthma prevalence only partially. The effects of lifestyle may be better understood through a combination of effects or a set of attributes allowing us to identify common features that may help explain differences in asthma prevalence between populations, and understand how aggregations of behaviours and personal characteristics shared by groups of individuals may alter disease risk [13].

In Latin America, asthma or wheeze prevalence has been associated with changes in lifestyles in urban and rural settings [11]. Nevertheless, there are no studies evaluating how differences in lifestyle affect the prevalence of asthma in both urban and rural areas. The present study identified common lifestyle domains in urban and rural populations of schoolchildren based on social and behavioural characteristics of the children and the households in which they live, and explore how these patterns could explain the prevalence of wheeze in a tropical region of Ecuador.

## Methods

### Study area and population

The study was conducted in urban and rural areas of a tropical region of Esmeraldas Province in northwest Ecuador. The population is predominantly Afro-Ecuadorian, although mestizo and indigenous populations are also present in this area. The rural study population comprised small communities in the Districts of San Lorenzo and Eloy Alfaro, located in the north of the province. Economic activities of these communities are focused on subsistence agriculture, hunting, fishing, logging, and more recently African palm oil cultivation. Commerce and the provision of services are available in the larger communities. The educational level of the population is generally very low with a high rate of adult illiteracy. Housing materials are a mixture of traditional and modern materials - wood and bamboo for walls and corrugated iron for roofing with the use of cement blocks for walls becoming increasingly common. Drinking water comes directly from rivers although some communities have untreated piped water sourced from streams. There is no sewage system in any of the rural communities and disposal of faeces is generally by household or community pit latrines or in the open. A number of communities are connected to the national electrical grid but few have access to telephone services or mobile networks. Transportation for most communities is by river, although small roads have been built to connect some of these communities with the provincial capital [14].

The urban study population was in Esmeraldas city, especially in neighbourhoods of Afro-Ecuadorian migrants coming from the districts of San Lorenzo and Eloy Alfaro (rural study population). Esmeraldas city is the provincial capital located ~100 km to the south of the rural study area. It is a medium sized coastal city with approximately 189,504 inhabitants, is the principal port in northwest Ecuador, the terminal for trans-Andean oil pipeline, and location of the country’s largest refinery. More than half of oil refined in Ecuador is refined in Esmeraldas, and most of the country’s oil exports are shipped from its port. Industrial facilities such as thermoelectric power plant (run on fuel oil processed by the refinery), a wood processing plant and the refinery are located in an area in the south of the city, being an important source of pollution for the city. The main economic and industrial activities are based on chemical and oil exports, commerce, agriculture (especially tropical fruit and palm oil), timber, fishing, and tourism. Basic services are of limited availability: 28% of the households have no potable water, 23% of the households lack a sewage system and 17% of the houses in city are constructed with traditional materials (wood and or bamboo). Extreme poverty is present in 21% of the population and educational level is low with just 10% of the population having 10 or more years of education [15].

### Study design

Cross-sectional studies were done in schoolchildren aged 5–16 years, in a convenience sample of 59 rural communities and 9 urban schools to evaluate lifestyle domains. The rural and urban schools were chosen as convenience samples to provide schools in which the children were predominantly Afro-Ecuadorian. The rural study was done in two rural Districts (Eloy Alfaro and San Lorenzo) in which small rural communities were selected each with a single school and <150 pupils on the school roll. In the urban area in the City of Esmeraldas, schools were selected from barrios or neighbourhoods in which there was a predominance of Afro-Ecuadorian migrants from the two northern rural Districts where the rural study was conducted. All children attending the schools at the time of the survey were eligible for inclusion. The response rate was 91.3% of children in rural schools and 90.8% of those attending urban schools (of the annually updated school lists). Selection of rural and urban Afro-Ecuadorian schoolchildren of similar ancestry should have minimized differences in lifestyle related to social and cultural differences between populations. Data collection for the rural population was done between March 2005 and May 2007 and for the urban population between November 2007 and January 2010. The original study was designed to investigate risk factors for allergy and asthma in Afro-Ecuadorian children associated with rural–urban migration and is described in detail elsewhere [16].

### Data collection

A parent or guardian of each child was interviewed by trained field workers using a questionnaire modified from the International Study of Asthma and Allergies in Childhood (ISAAC) phase II study [17] and adapted to the local conditions and translated into Spanish. We used recent wheeze as a proxy for asthma (defined as the presence of wheeze in the last 12 months), a definition that has been validated in different populations and that is widely used in epidemiologic studies [17, 18], and is especially useful in populations with limited access to health care [9]. Indicators to evaluate lifestyle were selected and classified into 4 variable groups (Table 1). 1) Socioeconomic status (SES) of the household, representing the socioeconomic position of the household in relation to the study population. We included variables for monthly household income and parental education and employment. 2) Characteristics of the child’s home, representing the type of housing in which the child lived including presence of basic services (electricity and running water), disposal of faeces (type of bathroom), electrical appliances, household construction materials and type of cooking fuel. 3) Sedentary characteristics of the child, representing the level of sedentarism including variables such as nutritional status (overweight vs. normal weight), consumption of fast foods (fizzy drinks and hamburgers), hours of daily TV viewing and frequency of vigorous physical exercise. Nutritional status was defined by z scores of body mass index (weight[kg]/height[m]^2^) to classify children as overweight (Z scores greater than or equal to the 85th centile) or of normal weight [19]. 4) Agricultural activities of the household, representing the child’s potential farming exposures including parental agricultural activities, contact with animals in farms and animal breeding in or around the household home. A fifth group of variables (Other asthma risk factors) representing other important risk factors for asthma including sex, age, maternal history of asthma, maternal smoking during pregnancy, environmental tobacco smoke exposure in the child’s household and household overcrowding were included also in the analyses (Table 1).

**Table 1:** **Characteristics of the study population by variable groups and area of residence**

Groups	Variables	Categories	Rural		Urban	
			n	%	n	%
Socioeconomic Status of the household	Father’s ^A^ education	<6 years	2512	58.5%	513	20.4%
		6-11 years	1395	32.5%	1268	50.5%
		>11 years	388	9.0%	729	29.0%
	Mother’s ^A^ education	<6 years	2444	56.9%	502	20.0%
		6-11 years	1498	34.9%	1296	51.6%
		>11 years	353	8.2%	712	28.4%
	Father’s employment	Farm worker	3356	78.1%	433	17.3%
		Employee	459	10.7%	1223	48.7%
		Trader	323	7.5%	507	20.2%
		Professional	157	3.7%	347	13.8%
	Mother’s employment	House wife	2880	67.1%	1254	50.0%
		Farm worker	610	14.2%	21	0.8%
		Employee	450	10.5%	735	29.3%
		Trader	195	4.5%	313	12.5%
		Professional	160	3.7%	187	7.5%
	Household income ^B^	≤$170	3523	82.0%	1077	42.9%
		$171-$340	627	14.6%	913	36.4%
		>$340	145	3.4%	520	20.7%
Characteristics of the child’s home	Basic Services	0-1 Services	4044	94.2%	210	8.4%
		2-3 Services	251	5.8%	2300	91.6%
	Source of drinking water	River/well	3052	71.1%	14	0.6%
		Piped	977	22.7%	185	7.4%
		Potable	266	6.2%	2311	92.1%
	House construction materials	Wood/Bamboo	2892	67.3%	710	28.3%
		Concrete/others	725	16.9%	638	25.4%
		Concrete	678	15.8%	1162	46.3%
	Bathroom	Field	1553	36.2%	97	3.9%
		Latrine	2742	63.8%	671	26.7%
		Toilet	0	0%	1742	69.4%
	Electrical appliances	0-2 appliances	2465	57.4%	417	16.6%
		3 appliances	1030	24.0%	943	37.6%
		4 appliances	800	18.6%	1150	45.8%
	Cooking fuel	Only gas	3074	71.6%	2355	93.8%
		Gas/wood/charcoal	1221	28.4%	155	6.2%
Sedentary characteristics of the child	Nutritional Status^C^	Normal weight	3786	88.1%	2069	82.4%
		Overweight	509	11.9%	441	17.6%
	Fizzy drink consumption	Sometimes	938	21.9%	843	33.7%
		1-4 times/ week	2476	57.8%	1293	51.7%
		>4 times/week	871	20.3%	364	14.6%
	Hamburger consumption	Never	3211	74.9%	968	38.8%
		Sometimes	718	16.7%	881	35.3%
		1 in a month	359	8.4%	649	26%
	Vigorous exercise	Daily	3197	74.7%	1862	74.2%
		1-3 times/week	970	22.7%	603	24%
		Sometimes	57	1.3%	17	0.7%
		Barely	55	1.3%	27	1.1%
	TV viewing	<1 hours daily	1136	26.4%	242	9.6%
		1-3 hours daily	2459	57.3%	1670	66.5%
		> = 4 hours daily	700	16.3%	598	23.8%
Groups	Variables	Categories	Rural		Urban	
			n	%	n	%
Agriculture activities of the household	Farm activities	No	851	19.8%	2071	82.5%
		Yes	3444	80.2%	439	17.5%
	Contact with animals in farms	No	2981	69.4%	2294	91.4%
		Yes	1314	30.6%	216	8.6%
	Pigs breeding around home	No	2203	51.4%	2104	83.8%
		Yes	2086	48.6%	406	16.2%
	Chicken breeding around home	No	644	15.0%	954	38.0%
		Yes	3648	85.0%	1554	62.0%
	Other farm animals around home	No	2523	58.8%	1789	71.3%
		Yes	1768	41.2%	719	28.7%
Others asthma risk factors	Sex	Male	2206	51.4%	1321	52.7%
		Female	2089	48.6%	1187	47.3%
	Age	<11 years	2224	51.8%	1689	67.3%
		≥11 years	2071	48.2%	821	32.7%
	Maternal asthma	No	3223	79.1%	1919	81.4%
		Yes	854	20.9%	439	18.6%
	Matrenal smoking during pregnancy	No	3720	88.2%	2300	94.2%
		Yes	499	11.6%	142	5.8%
	Environmental tobacco smoke^D^	No	2297	53.7%	1600	63.8%
		Yes	1981	46.3%	909	36.2%
	Household overcrowding^E^	≤3	2670	62.2%	1578	62.9%
		>3	1625	37.8%	932	37.1%

^A^<6 years = Incomplete primary or ilitare; 6-11 years = complete primery and incomplete secundary; >11 = complete secundary or higher. ^B^Household income based on the basic family wage in 2007 (US$170). ^C^Defined by z scores of body mass index (weight[kg]/height[m]^2^) to classify children as overweight (Z scores greater than or equal to the 85th centile [16]) or of normal weight. ^D^Exposure to a smoker living in the child’s household. ^E^Persons per sleeping room.

### Statistical analysis

Multiple correspondence analyses (MCA) is a technique that allows researchers to use a multivariate approach to lifestyle studies. MCA [20] is a descriptive, exploratory technique designed to analyse the pattern of relationships of several categorical dependent variables in order to produce a graphic illustration of the original information in a low dimensional space. MCA summarizes a set of categorical variables into a small number of orthogonal variables named “dimensions” that are related to the original variables through a pair of descriptive measures, discrimination and inertia. Discrimination measures show the correlation (-1 to 1) of each variable with a dimension, and inertia measures the proportion of variance of the original variables explained by each dimension. Dimensions produce a set of coordinate values (Z scores) that summarise subjects and categories permitting the associations between subjects and between categories to be displayed. Dimensions form the axes of the graphical representation of the associations between categories or subjects that can be visualized as clusters or patterns. A high proximity between categories represents a high level of association.

To identify lifestyle domains, we developed four models using MCA, one for each variable group (but not for the fifth group – Other asthma risk factors). We retained two dimensions in each model, evaluating measures of discrimination and inertia for each dimension. Graphical displays of the variable categories and subjects (children) were constructed using two dimensions for each variable group (i.e. by building a series of two-dimensional graphs). Graphs were evaluated to identify visually prominent lifestyle domains based on associations between variable categories. Having identified lifestyle patterns, we used non-hierarchical cluster analysis to allocate individual subjects to domains using Z scores for each subject [21]. Scatter plots were constructed to see how cluster analysis assigned children to lifestyle domains and were compared visually with those for the four variable categories that were built for each of the 4 MCA models. Similarly, cross-tabulation frequencies between domains and original variables were done to assess the distributions of each variable within each domain.

Unadjusted and multivariable logistic regression was used to evaluate the associations between lifestyle domains and wheeze prevalence. For multivariate logistic regression analyses we constructed models for rural and urban areas using the 4 lifestyle domains and other asthma risk factors (fifth variable group). The final models were selected using back-wards step-wise regression and were those that explained the most variation in wheeze prevalence, those with the smallest mean square error, and the highest value of adjusted R^2^. Distributions of variable categories by area of residence were evaluated using the Chi-squared test. Population-attributable fractions (PAFs) were calculated for each pattern [(proportion of exposed cases) × (OR -1)/OR], using adjusted ORs. Statistical significance was inferred by p < 0.05. All the analyses were done with SPSS version 18 (IBM SPSS, New York, USA).

The ethics committee of the Hospital Pedro Vicente Maldonado, Ecuador, approved the study protocol. Written informed consent was obtained from a parent and signed minor assent from the child.

## Results

We evaluated a total of 6,805 children of whom 4,295 lived in the rural area and 2,510 in the urban area. The prevalence of wheeze was similar in rural and urban schoolchildren (10.3% vs. 9.4%, respectively) with an overall prevalence of 9.7%. Characteristics of the study populations by variable groups and area are shown in Table 1. The frequencies of almost all variables were significantly different between urban and rural areas (p < 0.05), except sex and overcrowding.

**Figure 1:**
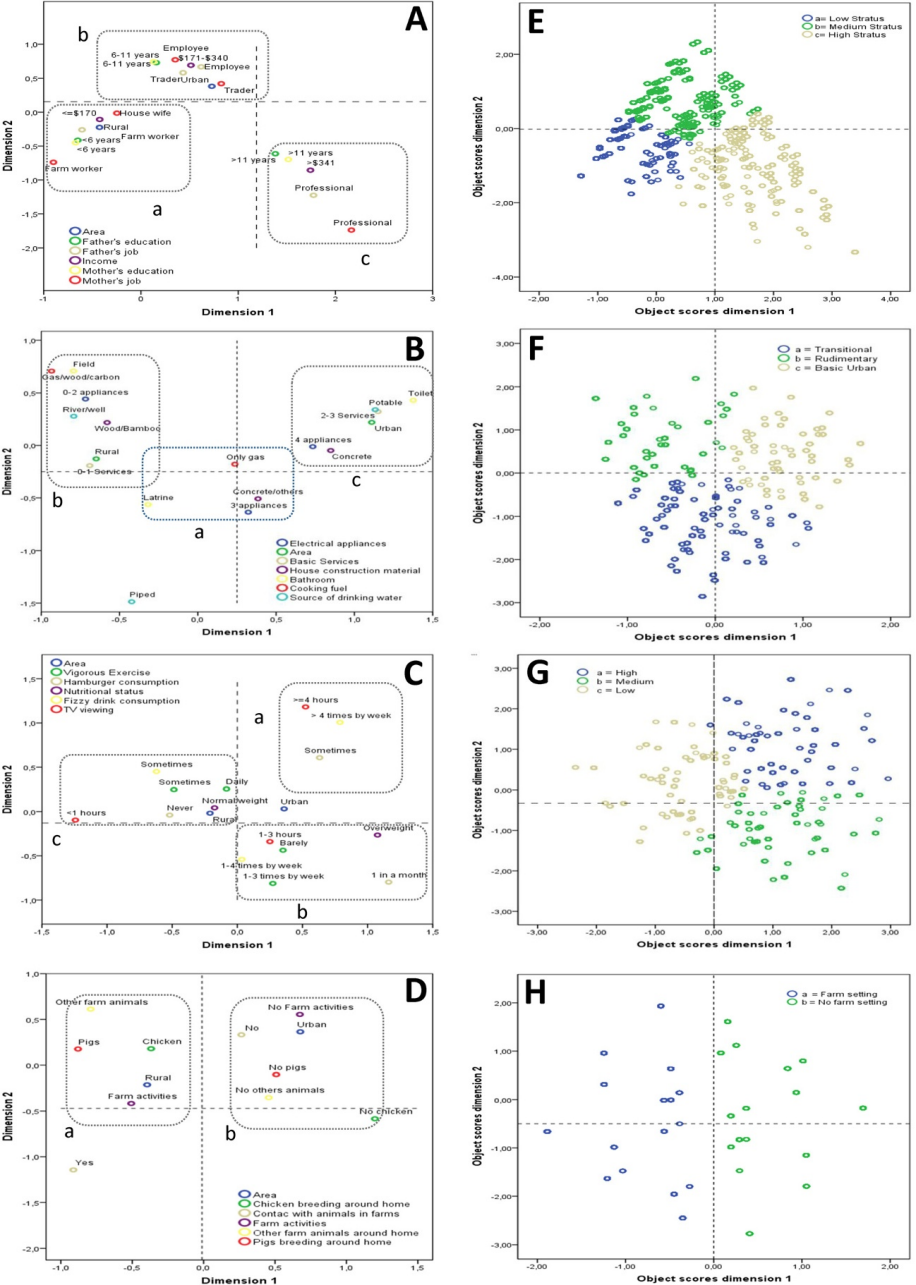
**Multiple Correspondence Analyses by:** **A)** Socioeconimc status of the household, **B)** Characteristics of the chikld’s home, **C)** Sedentary characteristics of the child, **D)** Agricultural activities of the household. Colour boxes around groupings of categories represent lifestyle domains. Axes represent z scoress by dimension. Cluster Analyses using objects scores of each MCA: **E)** Socioeconimc status of the household, **F)** Characteristics of the child’s home, **G)** Sedentary characteristics of the child, **H)** Agriculture activities of the household. Colour circles represent the number of children in each lifestyle domains.

Graphic presentations of the two dimensions obtained by MCA for each of the 4 variable groups are shown in Figures 1A-D. Discrimination measures and the proportion of variance accounted for by each dimension are provided in Additional file 1: Table S1. The model for socioeconomic status (SES) of the household identified three domains (Figure1A): a) Low SES - associated with a rural setting and represented by farm workers, low income (<=$170 or lower than the basic family wage in 2007) and low parental educational level (<6 years or incomplete primary education); b) Medium SES - associated with an urban setting represented by trade and non-professional employment, 6–11 years of education (or incomplete secondary education) and monthly income of $171-$340; c) High SES - represented by professional occupations, higher parental educational level (>11 years or complete secondary or higher level education) and income of > $340 (or more than 2 basic family wages). The model for characteristics of the child’s residence identified three domains (Figure 1B): a) Transitional - represented by presence of 3 electric appliances, gas for cooking, latrine bathroom and use of concrete and wood in house construction; b) Rudimentary - associated with rural areas and characterized by 1 or fewer basic services, less than 2 electric appliances, defaecation in the open, use of river or well water for drinking and use of wood or bamboo in house construction; c) Basic urban - associated with urban residence and represented by a household connection to potable water, concrete building materials for walls, use of a flushing toilet, ownership of a set of appliances and 2 or 3 basic services. The model for Sedentary characteristics of the child identified three domains (Figure 1C): a) High sedentarism – represented by consuming fizzy drinks > 4 times by week, watching television ≥ 4 hours daily and consumption of hamburgers sometimes; b) Medium sedentarism represented by vigorous physical activity 1–3 times per week, watching television 1–3 hours daily, consumption of fizzy drinks 1–4 times weekly, being overweight and consuming hamburgers once month; c) Low sedentarism - associated with rural settings and characterized by never consuming fizzy drinks and hamburgers, watching television <1 hour daily, normal weight and daily vigorous physical exercise. The model for agricultural activities of the household (Figure 1D) identified two domains: a) Farming - associated with rural settings and characterized by farm activities and peri-domestic animal breeding; b) Non-farming - associated with urban settings and represented by no farm activities, no animal husbandry around the household and no contact with farm animals. Cluster analysis was used to allocate the children to domains for each of the 4 variable groups. Scatter plots derived from these cluster analyses show the distributions of children by lifestyle domains in which dots represent individual children and dot colour represents lifestyle domains for each of the 4 variable groups (Figures 1E-H). Frequencies of original variables stratified by each domain for each model are shown in Additional file 1: Tables S2-S5 to illustrate the relative distributions of variable categories between domains. Low SES status (64.1%), rudimentary residence (51.6%), low sedentarism (67.9%) and farming environment (61.5%) were more common among rural school children (Table 2), while medium SES status (63.9%), basic urban type of residence (87.3%), low sedentarism (48.2%) and non-farming environment (85.3%) were more common among urban children (Table 2).

**Table 2:** **Distributions of children in lifestyle domains and prevalence of wheeze by study area**

Groups	Lifestyle Domains	Rural		Wheeze	Urban		Wheeze
		n	%	%	n	%	%
Socioeconomic Status of the household	Low	2751	64.1%	10.1%	231	9.2%	14.8%
	Medium	1255	29.2%	10.4%	1603	63.9%	8.7%
	High	289	6.7%	11.4%	676	26.9%	9.1%
Characteristics of the child’s home	Transitional	1860	43.3%	10.8%	257	10.2%	13.9%
	Rudimentary	2217	51.6%	10.3%	63	2.5%	15.8%
	Basic Urban	218	5.1%	5.7%	2190	87.3%	8.7%
Sedentarism of the child	High	584	13.6%	14.4%	533	21.2%	9.6%
	Medium	793	18.5%	10.7%	766	30.5%	8.9%
	Low	2918	67.9%	9.4%	1211	48.2%	9.6%
Agricultural activities of the household	Farming	2642	61.5%	10.1%	369	14.7%	12.4%
	Non-farming	1653	38.5%	10.7%	2141	85.3%	9.4%

There was evidence of differences in the prevalence of recent wheeze by lifestyle patterns for the four lifestyle variable groupings, and estimates of effect from univariate and multivariate analyses are shown in Table 3. Univariate analyses showed: 1) socioeconomic status of the household - a higher risk of wheeze in children of low versus high socioeconomic status in the urban but not the rural sample (interaction p = 0.026); 2) Characteristics of the child’s home - living in a basic urban house compared to a transitional or rudimentary house provided protection against wheeze in both urban and rural children; 3) sedentarism of the child – a high compared to low level of sedentarism was associated with an increased risk of wheeze in rural but not urban children (interaction p = 0.027); 4) agricultural activities of the household – farming compared to non-farming exposures were associated with wheeze in the urban but not rural samples (interaction P = 0.032).

**Table 3:** **Associations between recent wheeze and lifestyle domains or other asthma risk factors**

		Rural						Urban						
Groups	Lifestyle domains/Categories	Univariate			Multivariate			Univariate			Multivariate			Interact. ^A^
		OR	95% CI	P value	OR	95% CI	P value	OR	95% CI	P value	OR	95% CI	P value	P value
Socioeconomic status of the household	Low vs High	0.88	0.59-1.30	0.518				1.74	1.10-2.74	0.016	1.61	0.99-2.63	0.054	0.026
	Medium vs High	0.90	0.60-1.37	0.628				0.96	0.70-1.32	0.796	0.88	0.62-1.23	0.451	0.819
Characteristics of the child’s home	Transitional vs Basic urban	2.01	1.10-3.66	0.023	2.02	1.1-3.73	0.024	1.70	1.15-2.50	0.007	1.36	0.90-2.05	0.143	0.645
	Rudimentary vs Basic urban	1.89	1.04-3.44	0.037	1.88	1.02-3.47	0.043	1.97	0.95-4.08	0.067	2.38	1.12-5.05	0.024	0.932
Sedentarism of the child	High vs Low	1.63	1.24-2.12	<0.001	1.64	1.23-2.18	0.001	0.99	0.70-1.41	0.957				0.027
	Medium vs Low	1.16	0.89-1.50	0.269	1.22	0.93-1.60	0.154	0.91	0.66-1.25	0.568				0.253
Agricultural activities of the household	Farming vs. non-farming	0.93	0.76-1.15	0.513				1.45	1.02-2.06	0.036				0.032
Other asthma risk factors														
Sex	Male vs Female	0.93	0.76-1.14	0.480				0.92	0.70-1.20	0.526				0.926
Age (years)	<11 vs ≥11	1.72	1.40-2.12	<0.001	1.81	1.46-2.24	<0.001	1.27	0.94-1.72	0.115	1.39	1.0-1.92	0.051	0.108
Maternal sthma	Yes vs No	3.04	2.46-3.76	<0.001	3.03	2.44-3.75	<0.001	3.47	2.58-4.66	<0.001	3.48	2.58-4.71	<0.001	0.473
Matrenal smoking during pregnancy	Yes vs No	1.04	0.76-1.41	0.826				1.82	1.12-2.96	0.016	1.86	1.12-3.08	0.016	0.054
Environmental tobacco smoke^B^	Yes vs No	1.01	0.82-1.23	0.945				1.07	0.81-1.42	0.631				0.726
Household overcrowding^C^	>3 vs ≤ 3	1.19	0.98-1.46	0.086				0.99	0.75-1.32	0.959				0.297

Shown are univariate and multivariate analyses with estimated odds ratios (OR) and 95% confidence intervals (95% CI).

^A^Interaction p value between urban and rural in univariate analyses. ^B^Exposure to a smoker living in the child’s household. ^C^Persons per sleeping room.

Multivariate analyses (Table 3) showed that in the rural area, children living in transitional and rudimentary houses had 2.02 and 1.88-fold greater risk of wheeze, respectively, compared to children living in basic urban houses, while children with a high level of sedentarism had 1.64-fold greater risk of wheeze than those with a low level of sedentarism and this risk increased depending to SES of the household: Low SES 1.39, Medium SES 1.80 and High SES 3.26 (Table 4). In the urban area, children living in rudimentary houses had 2.38-fold greater risk of wheeze compared with those living in basic urban houses.

**Table 4:** **Associations between recent wheeze and sedentarism domains stratified for each level of socioeconomic domains among rural schoolchildren**

Socioeconomic status domains	Domains of Sedentarism	Rural population		
		OR	95% CI	p
Low SES	High vs Low	1.39	0.95-2.04	0.095
	Medium vs Low	1.48	1.07-2.04	0.017
Medium SES	High vs Low	1.80	1.15-2.81	0.011
	Medium vs Low	0.77	0.45-1.32	0.341
High SES	High vs Low	3.26	1.24-8.55	0.016
	Medium vs Low	1.27	0.47-3.43	0.634

Shown are multivariate analyses with estimated odds ratios (OR) and 95% confidence intervals (95% CI).

ORs also adjusted for age, characteristics of the child’s home, and agricultural activities.

The findings for univariate and multivariate associations between recent wheeze and other asthma risk factors are shown in Table 3. In multivariate analyses, children aged less than 11 years (versus ≥11 years) and children of mothers with a history of asthma had 1.81 and 3.03-fold greater risk of wheeze, respectively. In the urban area, children with a maternal history of asthma and of mothers who smoked during pregnancy had a 3.48 and 1.86-fold greater risk of wheeze, respectively.

We estimated the population fractions of wheeze attributable to each of the lifestyle domains (Table 5). In rural areas, transitional or rudimentary (versus basic urban) housing explained 22-24% of wheeze and high and medium versus low sedentarism accounted for 6% and 3% of wheeze, respectively. In contrast, lifestyle domains seemed to have poor explanatory power among urban schoolchildren. We stratified also the four lifestyle domains by sex and age groupings (<11 years versus ≥11 years) (see Additional file 1: Tables S6 and S7): in the rural area, associations between high sedentarism and wheeze were stronger in males and older children, while in urban areas, associations between housing characteristics and wheeze were stronger in females and in younger children.

**Table 5:** **Population fractions (PAF%) of recent wheeze attributable to each lifestyle domain**

Groups	Lifestyle Domain	Rural			Urban		
		Exposed cases	OR ^B^	PAF %	Exposed cases	OR ^B^	PAF %
Socioeconomic Status of the household	Low vs. High	0.64	0.88	<0	0.09	1.74	4%
	Medium vs. High	0.29	0.90	<0	0.64	0.96	<0
Characteristics of the child’s home	Transitional vs. Basic urban	0.43	2.01	22%	0.10	1.70	4%
	Rudimentary vs. Basic urban	0.52	1.89	24%	0.03	1.97	2%
Sedentarism of the child	High vs. Low	0.14	1.63	5%	0.21	0.99	<0
	Medium vs. Low	0.19	1.16	3%	0.31	0.91	<0
Agricultural activities of the household	Farming vs Non-farming	0.62	0.93	<0	0.15	1.45	5%

PAF% - populationa attribuatble fraction estimated using the proportion of exposed cases for each lifestyle pattern **//** OR^B^ = univariate odds ratios.

## Discussion

Lifestyle represents a complex constellation of highly associated factors involving social, behavioural and economic activities in which humans are engaged and environmental factors to which they are exposed. Lifestyle is in a constant state of change as human societies evolve, particularly under the influences of globalisation and increasing urbanization of humans worldwide – even the remotest of human populations have been strongly influenced by these processes. It has been extremely difficult for epidemiologists interested in the effects of lifestyle factors on the prevalence of wheeze and asthma to disentangle independent effects of individual risk factors that together constitute lifestyle. The approach adopted in the present analysis has been to define lifestyle as a set of attributes representing groups of linked risk factors rather than the traditional approach of investigating independent effects of individual risk factors [13]. In the present study, we used multiple correspondence analyses to identify key lifestyle domains and then cluster analysis to allocate individuals to specific lifestyle domains to explore how these patterns relate to the risk of wheeze in a large sample of school children living in urban and rural areas of tropical Ecuador. Our data provide evidence that factors associated with sub-standard and poor housing (i.e. lack of basic services including electricity, traditional construction materials and cooking fuel) may be an important determinant of the risk of wheeze overall while among generally highly physically active rural schoolchildren with generally high levels of activity, those with a high levels of sedentarism (low physical activity, overweight, and consumption of junk foods) are at greatest risk. Our data provide further insights into how groups of risk factors relating to lifestyle patterns may affect the risk of wheeze in a population undergoing the rapid transition from a traditional to a more modern and urban way of life.

Previous epidemiological studies have reported associations between prevalence of wheeze/asthma and lifestyle factors related to urbanization [5, 10, 12] although causal risk factors and mechanisms have not been clearly identified. These associations are often inconsistent between studies of urban and rural populations and between regions at different levels of development. For example, studies conducting in Zimbabwe and Ghana observed a higher prevalence of wheeze/asthma among urban children of higher SES [22, 23], while studies of children in urban areas of Latin America have reported a higher prevalence among the poor [11, 24]. In industrialized countries, associations between low SES and childhood asthma severity has been observed in different international studies [25–27]. Risk factors such as sedentary habits, an increase in the consumption of ‘junk’ food, and obesity have been identified as possible explanations for the increasing asthma prevalence in both developing and developed countries [28, 29], nevertheless some studies found no association between overweight/obesity and asthma trends [30]. Traditional lifestyles, especially those related to farming, have been postulated to provide protection against allergic diseases and atopy [5], and explain, at least in part, the lower prevalence in rural areas [4]. However, not all farming environments protect against childhood wheeze/asthma [31, 32]. It has been hypothesized that changes in housing construction creating a more artificial living environment might predispose to growth of indoor allergens affecting the occurrence of asthma in developed countries [33]. Housing with excessive moisture and dampness, inadequate or poorly maintained heating and ventilation systems, crowding, pest infestations, deteriorated carpeting, and structural defects may increase exposure to asthma triggers [34].

Our data showed that characteristics of the child’s household were associated with the prevalence of wheeze in both urban and rural areas. Children living in rudimentary and transitional households had a higher risk. In urban areas, there were trends of increased prevalence of wheeze in poor households and those with agricultural exposures. Our findings are consistent with a previous study done in City of Esmeraldas 20 years ago, which showed an association between low socioeconomic status and asthma prevalence, especially for those socioeconomic factors relating to housing materials [35]. Other studies from urban centres in Latin America have also shown associations between wheeze/asthma and risk factors indicative of poverty, dirt and infections [11, 24, 32]. In rural areas of our study, wheeze was more prevalent in children with a high level of sedentarism. Similarly, data from elsewhere have shown that an association between diet (and ‘junk’ food consumption) and asthma prevalence [28, 36].

In a previous ecologic study of the same rural communities analysed here, we showed that factors associated with greater socioeconomic level and changes towards a more urban lifestyle were associated with the community prevalence of childhood asthma [37]. The same ecologic analysis showed an association between community SES and wheeze prevalence. Our data here indicate that SES, in fact, may be acting as a confounder at the individual level. In the rural area, SES was strongly associated with the level of consumption and acculturation: a household needs an electrical connection to watch TV and TV viewing influences the level of consumption, while traditional exercise patterns will be modified by living close to a school and not having to fetch water because of a household water connection. In fact, our data showed that children allocated to the high SES and high sedentarism domains in rural areas had 3.26 greater risk of wheeze compared to children of high SES and low sedentarism.

Treating lifestyle as a set of attributes or domains allows us to understand better how urbanization processes may affect the prevalence of wheeze in rural and urban areas of a developing country. In transitional societies, it is common to find rural lifestyles co-existing in urban areas, particularly at the poor peripheries of cities that lack many basic services and where rural migrants settle and gradually become acculturated through the gradual adoption of modern habits and behaviours although remaining socially marginalized. This phenomenon could be observed in our urban study population by the proportion of households that retained agricultural activities (14.7% were classified as having farming attributes) and that lived in transitional and rudimentary households (10.8% & 10.3%, respectively). In urban areas, maintaining a traditional way of life is commonly associated with poverty and an increased risk for a number of diseases [38]. In the present study, there was evidence in the urban area for an effect of poverty on wheeze prevalence for domains within 3 of the 4 variable groups (e.g. low socioeconomic level, transitional housing, and farming exposures). This evidence is supported by the fact that severe asthma, which is less susceptible to misclassification of asthma, is more common among the poor, a pattern observed in epidemiological studies done in different geographical regions [25, 26]. On the other hand, urban influences in rural areas have led to the adoption of ‘modern’ behaviours, patterns of consumption, diet, and housing – seen in the rural area of the present study by the proportions of children with sedentary lifestyles (18.5% with medium and 13.6% with high sedentarism) and the proportion of children living in transitional homes (43.3%). Some components of the urbanization process have been clearly identified as detrimental for human health, especially asthma, such as consumption of junk food and the acquisition of sedentary habits [28, 36]. Nevertheless, other components of the urbanization process have been related to a reduction in the burden of disease through benefits gained by the provision of basic services and improvements in housing [39]. Our data indicate that a child living in better quality housing (termed basic urban) had a lower risk of wheeze than those living in rudimentary and transitional households.

Different lifestyle attributes may have distinct effects on the risk of wheeze in urban and rural populations. The level of development of each attribute and the speed by which they change in an relentless (and unstoppable) process of increasing urbanization could explain at least partly the increasing prevalence of wheeze/asthma that has been documented in some developing countries [3], and the declining disparity in prevalence between urban and rural areas [6, 7]. Furthermore, lifestyle factors probably operate at several levels on the risk of asthma such as individual, family, and community levels (i.e. contextual) – future studies addressing this question should collect data at all these levels and use a multilevel approach to understand better the differences in the observed prevalence of wheeze/asthma prevalence by geography and wealth [40].

The methodological limitations of our study include its cross-sectional design and the potential recall bias using questionnaire data. Furthermore, we restricted the study to mainly Afro-Ecuadorian children - until the findings are replicated in other populations, we cannot conclude their generalizability. Another limitation is that not all wheezing is asthma. Nonetheless, this definition, based on a symptom questionnaire, has been shown to distinguish between asthmatics and non asthmatics and has advantages in terms of cost, convenience, and the resulting optimization of sample sizes and response rates [17, 18]. Also, this definition is more practical in rural areas where access to health care is limited and use of doctor diagnosis may be subject to significant bias because of differential access to health care between populations. There was time period of up to 5 years between the start of data collection in the rural study population and completion of data collection in the urban area. It is possible that lifestyle exposures might start to change within such period in the two populations but we do not believe that such changes would be marked over such a relatively short period and that significant changes occur over decades rather than years. Finally, lifestyle involves a range of influences determined by cultural, economic and environmental factors, among others, that are hard to define and measure; including all such factors in an analysis is difficult. We approached this problem by selecting urban and rural populations that share a common history, culture and geography, and choosing common indicators based on asthma literature, sociological approach [37] and information available in our databases. Despite the fact that we collected detailed information related with lifestyle, other data that are likely to be important (eg, environmental sampling for air pollution, seasonal or geographical data) were not available. Smoking was not considered as a modern lifestyle indicator because smoking of tobacco (using ‘cachimbas’) in the rural area is a widespread and traditional practice, in contrast to the urban area where modern cigarettes are smoked. We did not collect data to distinguish smoking of traditional versus modern cigarette. In fact, the rural population had a higher proportion of mothers who smoked during pregnancy and household exposure to environmental tobacco smoke compared with the urban population (Table 1).

In conclusion, we have used a novel analytic approach to explore the effects of lifestyle domains or patterns on the prevalence of recent wheeze in schoolchildren living in rural and urban areas of Ecuador. Our data provide evidence that lifestyle domains relating to poverty (e.g. low socioeconomic status and living in transitional housing) are associated with a greater risk of wheeze in urban areas, and domains related to greater sedentarism and living in substandard housing are associated with a greater prevalence of wheeze in rural areas. These data indicate that different components of lifestyle may have distinct effects on the risk of wheeze between (i.e. rural vs. urban) and within populations at different stages of urbanization.

## Electronic supplementary material

Additional file 1: Table S1: Discrimination measures (dimensions) and proportion of variance explained for each dimension by variable groups. **Table S2.** Socioeconomics status of the household: variables stratified by domains. **Table S3.** Characteristics of the child’s home: variables stratified by domains. **Table S4.** Sedentary characteristics of the child: variables stratified by domains. **Table S5.** Agricultural activities of the household: variables stratified by domains. **Table S6.** Estimates odds ratios and 95% confidence intervals of recent wheeze for lifestyle domains controled by age group in urban and rural areas. **Table S7.** Estimates odds ratios and 95% confidence intervals of recent wheeze for lifestyle domains controled by sex in urban and rural areas. (DOCX 56 KB)

## References

[1] Woolcock AJ, Peat JK (1997). Evidence for the increase in asthma worldwide. Ciba Fousnd Symp.

[2] The international Study of Asthma and Allergies in Childhood (ISAAC) (1998). “Worldwide variation in prevalence of symptoms of asthma, allergic rhinoconjunctivitid, and atopic eczema. ISSAC. Lancet.

[3] Pearce N, Ait-Khaled N, Beasley R, Mallol J, Keil U, Mitchell E (2007). Worldwide trends in the prevalence of asthma symptoms: phase three of the International Study of Asthma and Allergies in Childhood (ISAAC). Thorax.

[4] Thomas A, Platts Mills E, Cooper P (2010). Differences in asthma between urban and rural communities in south Africa and other developing countries. J Allergy Clin Immunol.

[5] Von Hertzen L, Haahtela T (2006). Disconnection of man and the soil: reason for the asthma and atopic epidemic?. Allergy.

[6] Addo-Yobo EO, Woodcock A, Allotey A, Baffoe-Bonnie B, Strachan D, Custovic A: **Exercise-induced bronchospasm and atopy in Ghana: two surveys ten years apart.** *PLoS Med* **4**(2)**:**e70. doi: 10.1371/journal.pmed.0040070 10.1371/journal.pmed.0040070 PMC1808098 17326711

[7] Calvert J, Burney PGJ (2003). Increase in prevalence of exercise induced bronchospasm in a rural and urban population of African school children. Curr Allergy Clin Immunol.

[8] Weinmayr G, Weiland SK, Björkstén B, Brunekreef B, Büchele G, Cookson WO (2007). Atopic sensitization and the international variation of asthma symptom prevalence in children. Am J Respir Critical Care Med.

[9] Moncayo AL, Vaca M, Oviedo G, Erazo S, Quinzo I, Rosmeire LF (2010). Risk factors for atopic and non-atopic asthma in a rural area of Ecuador. Thorax.

[10] Weinberg EG (2000). Urbanization and childhood asthma: an African perspective. J Allergy Clin Immunol.

[11] Cooper PJ, Rodrigues L, Cruz A, Barreto ML (2009). Asthma in Latin America: a public health challenge and research opportunity. Allergy.

[12] VonHertzen L, Haahtela T (2004). Asthma and atopy the price of affluence?. Allergy.

[13] Frohlich K, Corin E, Potvin L (2001). A theoretical proposal for the relationship between context and disease. Sociol Health Illn.

[14] Sistema de Indicadores Sociales del Ecuador (2001) Accessed 25 August 2010 http://www.siise.gob.ec

[15] 15.INEC Instituto Ecuatoriano de Estadísticas y Censos (2010). Accessed 5 Jannuary 2015 http://www.inec.gob.ec

[16] Cooper PJ, Chico ME, Vaca MG, Rodriguez A, Alcantara-Neves N, Genser B (2006). Risk factors for asthma and allergy associated with urban migration: background and methodology of a cross-sectional study in Afro-Ecuadorian school children in Northeastern Ecuador (Esmeraldas-SCAALA Study). BMC Pulm Med.

[17] Weiland SK, Bjorksten B, Brunekreef B, Cookson WO, von Mutius E, Strachan DP (2004). Phase II of the International Study of Asthma and Allergies in Childhood (ISAAC II): rationale and methods. Eur Respir J.

[18] Asher MI, Keil U, Anderson HR, Beasley R, Crane J, Martinez F (1995). International Study of Asthma and Allergies in Childhood (ISAAC): rationale and methods. Eur Respir J.

[19] WHO Anthro Plus for personal computers Manual (2009). Software for assessing growth of the world’s children and adolescents.

[20] Carvalho H (2008). Analise Multivariada de dados Qualitativos, Utilizacao da analise de Correspondencias Multiplas com o SPSS.

[21] Guinot C, Latreille J, Malvy D, Preziosi P, Galan P, Hercberg S (2001). Use of multiple correspondence analysis and cluster analysis to study dietary behaviour: food consumption questionnaire in the SU.VI.MAX. cohort. Eur J Epidemiol.

[22] Keeley DJ, Neill P, Gallivan S (1991). Comparison of the prevalence of reversible airways obstruction in rural and urban Zimbabwean children. Thorax.

[23] Addo Yobo EO, Custovic A, Taggart SC, Asafo_Agyei AP, Woodcock (1997). Exercise induced broncho spasm in Ghana: differences in prevalence between urban and rural schoolchildren. Thorax.

[24] Barreto ML, Cunha SS, Fiaccone R, Esquivel R, Amorim LD, Alvim S (2010). Poverty, dirt, infections and non-atopic wheezing in children from a Brazilian urban center. Respir Res.

[25] Bråbäck L, Hjern A, Rasmussen F (2005). Social class in asthma and allergic rhinitis: a national cohort study over three decades. Eur Respir J.

[26] Mielck A, Reitmeir P, Wjst M (1996). Severity of childhood asthma by socioeconomic status. Int J Epidemiol.

[27] Littlejohns P, Macdonald L (1993). The relationship between severe asthma and social class. Respir Med.

[28] Ellwood P, Asher MI, García-Marcos L, Williams H, Keil U, Roberstson C (2013). Do fast foods cause asthma, rhinoconjunctivitis and eczema? Global findings from the International Study of Asthma and Allergies in Childhood (ISAAC) Phase Three. Thorax.

[29] Figueroa-Munoz J, Chinn S, Rona R (2001). Association between obesity and asthma in 4–11 year old children in the UK. Thorax.

[30] Chinn S, Rona R (2001). Can the increase in body mass index explain the rising trend in asthma in children?. Thorax.

[31] Ege MJ, Frei R, Bieli C, Schram-Bijkerk D, Waser M, Benz MR (2007). Not all farming environments protect against the development of asthma and wheeze in children. J Allergy Clin Immunol.

[32] Cooper PJ, Vaca M, Rodriguez A, Chico ME, Santos DN, Rodrigues LC (2013). Hygiene, atopy and wheeze–eczema–rhinitis symptoms in school children from urban and rural Ecuador. Thorax.

[33] Maziak W (2005). The asthma epidemic and our artificial habitats. BMC Pulm Med.

[34] Krieger J (2010). Home is where the triggers are: Increasing asthma control by improving the home environment. Paediatr Allergy Immunol Pulm.

[35] Harari R, Forastiere F, Vaca A, Bernal J, Caicedo J, Gaspar C, Comba P, Harari R (2004). Pobreza y otros factores de riesgo para el asma y sibilancias entre niños afroecuatorianos. El Ambiente y la Salud, Epidemiología ambiental. Primera edición.

[36] Hijazi N, Abalkhail B, Seaton A (2000). Diet and childhood asthma in a society in transition: a study in urban and rural Saudi Arabia. Thorax.

[37] Rodriguez A, Vaca M, Oviedo G, Erazo S, Chico ME, Teles C (2011). Urbanisation is associated with prevalence of childhood asthma in diverse, small rural communities in Ecuador. Thorax.

[38] Montgomery M, Ezeh A, Galea, Vlahov D (2005). The Health of Urban Populations in Developing Countries. Handbook of urban health.

[39] Barreto ML, Genser B, Strina A, Teixeira MG, Assis AM, Rego RF (2010). Impact of a Citywide Sanitation Program in Northeast Brazil on Intestinal Parasites Infection in Young Children. Environ Health Perspect.

[40] Wright RJ, Fischer EB, Kwachi I, Berkman LF (2003). Putting Asthma into Context: Community Influences on Risk, Behavior, and Intervention. Neighbourhoods and Health.

