# Supplementary material for: Lifestyle domains as determinants of wheeze prevalence in urban and rural schoolchildren in Ecuador: cross sectional analysis
Source: Environ Health. 2015 Feb 4;14:15. doi: 10.1186/1476-069X-14-15 (PMC4417196; doi:10.1186/1476-069X-14-15)
Supplement: Supplementary file 1 — Additional file 1: Table S1: Discrimination measures (dimensions) and proportion of variance explained for each dimension by variable groups. Table S2. Socioeconomics status of the household: variables stratified by domains. Table S3. Characteristics of the child’s home: variables stratified by domains. Table S4. Sedentary characteristics of the child: variables stratified by domains. Table S5. Agricultural activities of the household: variables stratified by domains. Table S6. Estimates odds ratios and 95% confidence intervals of recent wheeze for lifestyle domains controled by age group in urban and rural areas. Table S7. Estimates odds ratios and 95% confidence intervals of recent wheeze for lifestyle domains controled by sex in urban and rural areas. (DOCX 56 KB) [file 12940_2014_849_MOESM1_ESM.docx]

**Table S1. Discrimination measures (dimensions) and proportion of variance explained for each dimension by variable groups.**

| **Groups** | **Variables** | **Dimensions** | | **Mean** |
| --- | --- | --- | --- | --- |
|  |  | **1** | **2** |  |
| **Socioeconomic**  **Status of the household** | Father's education | 0.510 | 0.345 | 0.428 |
|  | Mother's education | 0.562 | 0.389 | 0.476 |
|  | Father's job | 0.555 | 0.300 | 0.428 |
|  | Mother's job | 0.478 | 0.321 | 0.372 |
|  | Household income | 0.478 | 0.086 | 0.333 |
|  | **% of Variance ^A^** | 50.59 | 30.82 | 40.71 |
| **Characteristics of the child’s home** | Basic Services | 0.794 | 0.062 | 0.428 |
|  | Drinking water source | 0.799 | 0.456 | 0.628 |
|  | House construction materials | 0.402 | 0.078 | 0.240 |
|  | Bathroom | 0.688 | 0.328 | 0.508 |
|  | Electrical appliances | 0.403 | 0.199 | 0.301 |
|  | Cooking fuel | 0.221 | 0.127 | 0.174 |
|  | **% of Variance ^A^** | 55.11 | 20.83 | 37.97 |
| **Sedentary**  **characteristic of the child** | BMI Z value | 0.188 | 0.011 | 0.100 |
|  | Soda | 0.214 | 0.400 | 0.307 |
|  | Hamburger | 0.460 | 0.182 | 0.321 |
|  | Exercise | 0.026 | 0.204 | 0.115 |
|  | TV viewing | 0.403 | 0.338 | 0.371 |
|  | **% of Variance ^A^** | 25.84 | 22.70 | 24.27 |
| **Agriculture activities of the**  **household** | Farm activities | 0.342 | 0.231 | 0.287 |
|  | Contact with animals in farms | 0.241 | 0.380 | 0.311 |
|  | Pigs breeding around home | 0.447 | 0.018 | 0.233 |
|  | Chicken breeding around home | 0.442 | 0.105 | 0.273 |
|  | Other farm animals around home | 0.361 | 0.216 | 0.288 |
|  | **% of Variance ^A^** | 36.64 | 19 | 27.82 |

**^A^ % variance represents the sum of inertias for each constituent variable belonging to each variable group.**

**Table S2. Socioeconomics status of the household: variables stratified by domains.**

|  | | **Domains** | | | | | |
| --- | --- | --- | --- | --- | --- | --- | --- |
| **Variables** | **Categories** | **Low SES** | | **Medium SES** | | **High SES** | |
|  |  | **n** | **%** | **n** | **%** | **n** | **%** |
| Father's education^A^ | <6 years | 2356 | 79.0% | 601 | 21.0% | 68 | 7.0% |
|  | 6-11 years | 488 | 16.4% | 1966 | 68.8% | 209 | 21.7% |
|  | >11 years | 138 | 4.6% | 291 | 10.2% | 688 | 71.3% |
| Mother's education^A^ | <6 years | 2385 | 80.0% | 498 | 17.4% | 63 | 6.5% |
|  | 6-11 years | 485 | 16.3% | 2095 | 73.3% | 214 | 22.2% |
|  | >11 years | 112 | 3.8% | 265 | 9.3% | 688 | 71.3% |
| Father's job | Farm worker | 2662 | 89.3% | 1006 | 35.2% | 121 | 12.5% |
|  | Employee | 174 | 5.8% | 1197 | 41.9% | 311 | 32.2% |
|  | Trader | 117 | 3.9% | 600 | 21.0% | 113 | 11.7% |
|  | Professional | 29 | 1.0% | 55 | 1.9% | 420 | 43.5% |
| Mother's job | House wife | 2171 | 72.8% | 1595 | 55.8% | 368 | 38.1% |
|  | Farm worker | 573 | 19.2% | 50 | 1.7% | 8 | 0.8% |
|  | Employee | 158 | 5.3% | 894 | 31.3% | 133 | 13.8% |
|  | Trader | 73 | 2.4% | 310 | 10.8% | 125 | 13.0% |
|  | Professional | 7 | 0.2% | 9 | 0.3% | 331 | 34.3% |
| Income^B^ | <=$170 | 2734 | 91.7% | 1608 | 56.3% | 258 | 26.7% |
|  | $171-$340 | 198 | 6.6% | 1106 | 38.7% | 236 | 24.5% |
|  | >$341 | 50 | 1.7% | 144 | 5.0% | 471 | 48.8% |

^A^ <6 years (incomplete primary); 6-11 years (incomplete secondary; >11 years (complete secondary and higher).

^B^Income is stratified by number of basic wages based on on a basic family income (canasta familiar) of US$170 in 2007.

**Table S3. Characteristics of the child’s home: variables stratified by domains.**

|  | |  | | **Domains** | |  | |
| --- | --- | --- | --- | --- | --- | --- | --- |
| **Variables** | **Categories** | **Transitional** | | **Rudimentary** | | **Basic Urban** | |
|  |  | **n** | **%** | **n** | **%** | **n** | **%** |
| Basic Services | 0-1 Services | 2017 | 95.3% | 2211 | 97% | 26 | 1.1% |
|  | 2-3 Services | 100 | 4.7% | 69 | 3% | 2382 | 98.9% |
| Source of drinking water | River/well | 899 | 42.5% | 2165 | 95% | 2 | 0.1% |
|  | Piped | 1119 | 52.9% | 28 | 1.2% | 15 | 0.6% |
|  | Potable | 99 | 4.7% | 87 | 3.8% | 2391 | 99.3% |
| House construction materials | Wood/Bamboo | 925 | 43.7% | 2003 | 87.9% | 674 | 28.0% |
|  | Concrete/others | 672 | 31.7% | 124 | 5.4% | 567 | 23.5% |
|  | Concrete | 520 | 24.6% | 153 | 6.7% | 1167 | 48.5% |
| Bathroom | Field | 288 | 13.6% | 1291 | 56.6% | 71 | 2.9% |
|  | Latrine | 1757 | 83.0% | 987 | 43.3% | 669 | 27.8% |
|  | Toilet | 72 | 3.4% | 2 | 0.1% | 1668 | 69.3% |
| Electrical appliances | 0-2 appliances | 623 | 29.4% | 1851 | 81.2% | 408 | 16.9% |
|  | 3 appliances | 889 | 42.0% | 240 | 10.5% | 844 | 35.0% |
|  | 4 appliances | 605 | 28.6% | 189 | 8.3% | 1156 | 48% |
| Cooking fuel | Only gas | 1872 | 88.4% | 1270 | 55.7% | 2287 | 95% |
|  | Gas/wood/charcoal | 245 | 11.6% | 1010 | 44.3% | 121 | 5% |

**Table S4. Sedentary characteristics of the child: variables stratified by domains.**

|  | |  | | **Domains** | |  | |
| --- | --- | --- | --- | --- | --- | --- | --- |
| **Variables** | **Categories** | **High** | | **Medium** | | **Low** | |
|  |  | **n** | **%** | **n** | **%** | **n** | **%** |
| BMI Z score | Normal weight | 850 | 76.1% | 1014 | 65.0% | 3991 | 96.7% |
|  | Overweight | 267 | 23.9% | 545 | 35.0% | 138 | 3.3% |
| Fizzy Drinks | Sometimes | 170 | 15.2% | 155 | 10.0% | 1456 | 35.4% |
|  | 1-4 times by week | 261 | 23.4% | 1274 | 82.0% | 2234 | 54.3% |
|  | > 4 times by week | 686 | 61.4% | 125 | 8.0% | 424 | 10.3% |
| Hamburger | Never | 260 | 23.3% | 621 | 39.9% | 3298 | 80.2% |
|  | Sometimes | 615 | 55.1% | 233 | 15.0% | 751 | 18.3% |
|  | once a month | 242 | 21.7% | 703 | 45.2% | 63 | 1,5% |
| Exercise | Daily | 922 | 82.6% | 623 | 40.0% | 3514 | 85.4% |
|  | 1-3 times per week | 168 | 15.1% | 882 | 56.7% | 523 | 12.7% |
|  | Sometimes | 16 | 1.4% | 5 | 0.3% | 53 | 1.3% |
|  | Barely | 10 | 0.9% | 46 | 3.0% | 26 | 0.6% |
| TV viewed (daily) | <1 hours | 21 | 1.9% | 58 | 3.7% | 1299 | 31.5% |
|  | 1-3 hours | 354 | 31.7% | 1430 | 91.7% | 2345 | 56.8% |
|  | >=4 hours | 742 | 66.4% | 71 | 4.6% | 485 | 11.7% |

**Table S5.** **Agricultural activities of the household: variables stratified by domains**

|  | | **Domains** | | | |
| --- | --- | --- | --- | --- | --- |
| **Variables** | **Categories** | **Farm environment** | | **Non-farming environment** | |
|  |  | **n** | **%** | **n** | **%** |
| Farm activities | No | 416 | 13.8% | 2506 | 66.1% |
|  | Yes | 2595 | 86.2% | 1288 | 33.9% |
| Contact with animals on farms | No | 1738 | 57.7% | 3537 | 93.2% |
|  | Yes | 1273 | 42.3% | 257 | 6.8% |
| Pigs breeding around house | No | 914 | 30.4% | 3393 | 89.5% |
|  | Yes | 2095 | 69.6% | 397 | 10.5% |
| Chicken breeding around house | No | 59 | 2.0% | 1539 | 40.6% |
|  | Yes | 2951 | 98.0% | 2251 | 59.4% |
| Other farm animals around house | No | 1051 | 34.9% | 3261 | 86.1% |
|  | Yes | 1959 | 65.1% | 528 | 13.9% |

**Table S6.** E**stimates odds ratios and 95% confidence intervals of recent wheeze for lifestyle domains controled by age group in urban and rural areas.**

|  |  | Rural | | | | | | Urban | | | | | |
| --- | --- | --- | --- | --- | --- | --- | --- | --- | --- | --- | --- | --- | --- |
| Lifestyle Domains | **Categories** | **<11** | | | **≥11** | | | **<11** | | | **≥11** | | |
|  |  | **OR** | **CI 95%** | **p** | **OR** | **CI 95%** | **p** | **OR** | **CI 95%** | **p** | **OR** | **CI 95%** | **p** |
| Socioeconomic  status of the  household | Low vs High | 0.85 | 0.52-1.41 | 0.534 | 0.93 | 0.49-1.78 | 0.832 | 1.76 | 1.01-3.07 | 0.045 | 1.69 | 0.76-3.75 | 0.196 |
|  | Medium vs High | 0.92 | 0.55-1.56 | 0.92 | 0.84 | 0.42-1.69 | 0.627 | 1.11 | 0.76-1.62 | 0.599 | 0.68 | 0.38-1.22 | 0.196 |
| Characteristics  of the child’s  home | Transitional  vs Basic urban | 2.19 | 1.12-4.28 | 0.022 | 2.65 | 0.64-11.1 | 0.180 | 1.72 | 1.09-2.71 | 0.019 | 1.61 | 0.76-3.40 | 0.213 |
|  | Rudimentary  vs Basic urban | 1.79 | 0.92-3.48 | 0.089 | 3.09 | 0.75-12.8 | 0.120 | 2.65 | 1.19-5.90 | 0.017 | 0.67 | 0.09-5.08 | 0.695 |
| Sedentarism  of the child | High vs Low | 1.35 | 0.95-1.93 | 0.095 | 2.21 | 1.47-3.32 | <0.001 | 1.02 | 0.68-1.54 | 0.920 | 0.96 | 0.48-1.90 | 0.899 |
|  | Medium vs Low | 1.19 | 0.86-1.67 | 0.298 | 1.22 | 0.80-1.85 | 0.364 | 0.8 | 0.55-1.17 | 0.251 | 1.25 | 0.71-2.23 | 0.442 |
| Agricultural activities  of the household | Farming vs.  non-farming | 0.90 | 0.70-1.17 | 0.430 | 1.03 | 0.73-1.44 | 0.883 | 1.32 | 0.86-2.04 | 0.204 | 1.83 | 1.02-3.32 | 0.049 |

ORs adjusted by: characteristics of the child’s home, sedentarism of the child, agricultural characteristics of the houshold and sex.

**Table S7.** E**stimates odds ratios and 95% confidence intervals of recent wheeze for lifestyle domains controled by sex in urban and rural areas.**

|  |  | Rural | | | | | | Urban | | | | | |
| --- | --- | --- | --- | --- | --- | --- | --- | --- | --- | --- | --- | --- | --- |
| Lifestyle Domains | **Categories** | **Male** | | | **Female** | | | **Male** | | | **Female** | | |
|  |  | **OR** | **CI 95%** | **p** | **OR** | **CI 95%** | **p** | **OR** | **CI 95%** | **p** | **OR** | **CI 95%** | **p** |
| Socioeconomic  status of the  household | Low vs High | 1.39 | 0.69-2.80 | 0.360 | 0.68 | 0.42-1.01 | 0.114 | 1.63 | 0.83-3.21 | 0.158 | 1.8 | 0.97-3.34 | 0.062 |
|  | Medium vs High | 1.66 | 0.81-3.41 | 0.170 | 0.59 | 0.35-1.0 | 0.05 | 1.09 | 0.70-1.71 | 0.704 | 0.84 | 0.53-1.31 | 0.434 |
| Characteristics  of the child’s  home | Transitional  vs Basic urban | 2.28 | 0.91-5.74 | 0.08 | 1.81 | 0.82-4.0 | 0.144 | 1.57 | 0.88-2.81 | 0.126 | 1.80 | 1.06-3.03 | 0.028 |
|  | Rudimentary  vs Basic urban | 1.95 | 0.78-4.89 | 0.157 | 1.90 | 0.86-4.17 | 0.113 | 1.14 | 0.34-3.80 | 0.837 | 3.12 | 1.22-7.98 | 0.017 |
| Sedentarism  of the child | High vs Low | 1.95 | 1.36-2.8 | <0.001 | 1.33 | 0.90-1.97 | 0.156 | 1.12 | 0.69-1.82 | 0.654 | 0.89 | 0.53-1.48 | 0.887 |
|  | Medium vs Low | 1.26 | 0.86-1.86 | 0.242 | 1.06 | 0.74-1.50 | 0.755 | 1.19 | 0.77-1.85 | 0.435 | 0.69 | 0.44-1.09 | 0.691 |
| Agricultural activities  of the household | Farming vs.  non-farming | 0.77 | 0.58-1.03 | 0.08 | 1.13 | 0.85-1.52 | 0.398 | 1.46 | 0.89-2.39 | 0.130 | 1.44 | 0.88-2.36 | 0.152 |

ORs adjusted by: characteristics of the child’s home, sedentarism of the child, agricultural characteristics of the houshold, and age.
